# Doctors, Nurses, Patients, and Lady Priestley

**Published:** 1897-01-09

**Authors:** 


### DOCTORS, NURSES, PATIENTS, AND LADY PRIESTLEY.

The modern nurse is evidently in evil case; her enemies throw scorn upon her, and, last of all, Lady Priestley fires off a philippic in the *Nineteenth Century* which surely should make the nurse *à la mode*, whoever she may be, hide her head in very shame.

It is stated that while some women undertake nursing "in the *bona-fide* hope of doing something to help suffering humanity, there are others who rush in for it in a pure spirit of adventure," and it is suggested that the adventures which may happen to them are decidedly various. We are told of "young and pretty women being found in the apartments of young and handsome men who for the time are enjoying bad health, and are not imbued with any wild desire for convalescence"; of "bachelors' apartments" to which "the door is thrown open, and a young woman in a costume not altogether unbecoming enters to mount guard day and night"; of "wives being intensely jealous of the woman installed in the husband's bed-chamber"; of "society" at a Continental health resort being "scandalised" at what Mrs. Grundy at home would have called the "goings on" of a young and pretty nurse who was there in sole attendance on a young English gentleman; and we are referred to the records of the Probate Court for proof of the "extraordinary influence which has occasionally been exercised by sick nurses over sick men in their last illnesses." We are told also of the "marriages which must necessarily spring from opportunities which present themselves all along the line of duty"—to such an extent, in fact, that an invidious world styles the new profession, "The new road to matrimony," and as showing how great these opportunities are we learn that "uncontrolled by vows, untroubled by austerity, the nurse of the period, guardian of the sick bed, and watcher over the solemn moments of expiring life, may be found taking part joyously in many of the frivolities around us." The moralities, however, are but one side of the question. The nurse *à la mode* knows too much, or thinks she does; she interferes with the diet ordered by the doctor, she criticises the treatment, she refuses to give the morphia prescribed by the physician, saying "She always threw it away and gave milk and water instead, which did just as well." She is masterful, and she is many other things besides.

At Christmas time, imbued with feelings of goodwill towards men—and towards women too—we trained our humble pen to write the nicest things it knew of concerning our good friends the nurses. It may be imagined, then, with what a shock this avalanche of hard sayings came upon us. But after all, sharp as is the onslaught, it is clearly the outcome of a widespread feeling of distrust of trained nurses as a class, a feeling which, while partly only a relic of old prejudices, undoubtedly is partly the result of the iron rule which some nurses are endeavouring to force upon their fellows in regard to uniformity of training and uniformity of status.

There are nurses who are veritable angels doing good on earth, but then there are some who are the opposite; there are nurses who are clever, trustworthy, and untiring, while there are others who are as flabby in body as they are silly in mind; yet all alike are trained nurses. They admit no difference in their fees or in their status, and when they join themselves together in co-operative association, they expect to take the cases in their turn as applications are sent in, quite irrespective of their suitability. This is a point to which we have several times drawn attention. What is every year becoming clearer is that it is not altogether the training that makes the nurse, and that, out of the crowds of women who enter for it, a large number can never attain to the first class. Why, then, not admit a second class both in capacity and in fees? At the present time neither the public nor the medical profession have any security that they will get a nurse really suited to the case however much they may pay, while people with slight ailments are deprived of the sick attendance they require by the fact that they must pay for a nurse trained for all eventualities if they are to have a nurse at all. It is like peeling a potato with a silver knife. "Surely," says Lady Priestley, "for a guinea a week an intelligent woman, after a minimum training . . . ought to understand the hygiene of the sick-room, know how to carry out the instructions of the doctor, how to make the bed, keep the room clean if necessary, &c." There can be no doubt that there is a great public demand for women of this kind, and that unless it can be arranged that they shall be admitted into the ranks of the nursing sisterhood, although as a second grade, they will hover around them and be a perpetual menace to their prosperity.

